# Computer-assisted determination of left ventricular endocardial borders reduces variability in the echocardiographic assessment of ejection fraction

**DOI:** 10.1186/1476-7120-6-55

**Published:** 2008-11-11

**Authors:** Eva Maret, Lars Brudin, Lena Lindstrom, Eva Nylander, Jan L Ohlsson, Jan E Engvall

**Affiliations:** 1 Department of Clinical Physiology, Ryhov County Hospital, SE-55185 Jonkoping, Sweden; 2 Center of Medical Image Science and Visualization, Linkoping University, SE-58185 Linkoping, Sweden; 3 Department of Clinical Physiology, Kalmar County Hospital SE-39185 Kalmar, Sweden; 4 Department of Medical and Health Sciences, Linkoping University Hospital, SE-58185 Linkoping, Sweden; 5 Department of Clinical Physiology, Vrinnevi Hospital, SE-60182 Norrkoping, Sweden; 6 Department of Clinical Physiology, Linkoping University Hospital, SE-58185 Linkoping, Sweden

## Abstract

**Background:**

Left ventricular size and function are important prognostic factors in heart disease. Their measurement is the most frequent reason for sending patients to the echo lab. These measurements have important implications for therapy but are sensitive to the skill of the operator. Earlier automated echo-based methods have not become widely used. The aim of our study was to evaluate an automatic echocardiographic method (with manual correction if needed) for determining left ventricular ejection fraction (LVEF) based on an active appearance model of the left ventricle (syngo^®^AutoEF, Siemens Medical Solutions). Comparisons were made with manual planimetry (manual Simpson), visual assessment and automatically determined LVEF from quantitative myocardial gated single photon emission computed tomography (SPECT).

**Methods:**

60 consecutive patients referred for myocardial perfusion imaging (MPI) were included in the study. Two-dimensional echocardiography was performed within one hour of MPI at rest. Image quality did not constitute an exclusion criterion. Analysis was performed by five experienced observers and by two novices.

**Results:**

LVEF (%), end-diastolic and end-systolic volume/BSA (ml/m^2^) were for uncorrected AutoEF 54 ± 10, 51 ± 16, 24 ± 13, for corrected AutoEF 53 ± 10, 53 ± 18, 26 ± 14, for manual Simpson 51 ± 11, 56 ± 20, 28 ± 15, and for MPI 52 ± 12, 67 ± 26, 35 ± 23. The required time for analysis was significantly different for all four echocardiographic methods and was for uncorrected AutoEF 79 ± 5 s, for corrected AutoEF 159 ± 46 s, for manual Simpson 177 ± 66 s, and for visual assessment 33 ± 14 s. Compared with the expert manual Simpson, limits of agreement for novice corrected AutoEF was lower than for novice manual Simpson (0.8 ± 10.5 vs. -3.2 ± 11.4 LVEF percentage points). Calculated for experts and with LVEF (%) categorized into < 30, 30–44, 45–54 and ≥ 55, kappa measure of agreement was moderate (0.44–0.53) for all method comparisons (uncorrected AutoEF not evaluated).

**Conclusion:**

Corrected AutoEF reduces the variation in measurements compared with manual planimetry, without increasing the time required. The method seems especially suited for unexperienced readers.

## Background

Left ventricular end-diastolic volume, ejection fraction (LVEF) and wall thickness are strong predictors for survival in most types of cardiac diseases. These measurements have important implications for therapy. The optimal timing for valve surgery and supportive surgery for heart failure such as ventricular restraint, ventricular restoration and left ventricular assist device surgery all rely upon measures of volume and LVEF, not to mention decisions on resynchronisation therapy, implantation of cardiac defibrillators and monitoring of anti-neoplastic drug treatment [1-4]. The measurement of cardiac volumes and ejection fraction should be accurate and reproducible, easy to use, affordable, non-invasive and without radiation exposure to the patient. Left ventricular size and function can be assessed by many modalities such as two- and three-dimensional echocardiography, myocardial scintigraphy (gated SPECT, equilibrium radionuclide angiography), contrast ventriculography, cardiac magnetic resonance and as of late also cardiac computed tomography. Two-dimensional echocardiography has, due to its ability to fulfil many of the requirements on the clinician's wish list, a central role in the clinical setting. It is widely used but demanding on the operator and sensitive to poor acoustic windows. The echocardiographic quantification method recommended by the European Society of Cardiology [5] is the biplane method of discs (modified Simpson's rule). This method requires manual tracing of the endocardial border in end-diastole and end-systole in two apical orthogonal planes. The method is time-consuming, requires visualization of the endocardial border of the entire left ventricular cavity and substantial expertise in positioning the patient correctly to avoid foreshortening of the long axis of the left ventricle (LV). Most frequently, quantification of LV function is performed by visual estimation and reports have claimed high accuracy in comparison with more objective methods, at least for trained observers [6,7]. However, a recent meta-analysis suggested a wide variability in this subjective assessment [8]. Measuring cardiac volumes and ejection fraction is basically a problem of segmentation, which requires the highest possible contrast-to-noise ratio between myocardium and blood pool. The earliest semi-automatic methods using ultrasound to detect the endocardial border (e.g. acoustic quantification and colour kinesis) were dependent on high quality images and optimal gain settings [9]. In clinical practice, overweight patients and those with obstructive pulmonary disease are difficult to scan and require the best imaging equipment. Several inventions have improved image quality, especially the use of harmonic imaging and external echo contrast. Harmonic imaging is now standard procedure, but cost and concerns about safety have prevented the widespread use of external echo contrast [10].

In studies, patients with expected non-visualization of the endocardial border are often excluded from assessment of LVEF [11]. One way of circumventing the problem with image quality is to focus on specific aspects of LV function such as long-axis function derived from measuring mitral annular motion (MAM). The relationship between LVEF and MAM is however complex and expresses the interaction between long- and short axis motion of the LV which changes with age and cardiac disease processes [12-15].

Considering the variation in the planimetry of the left ventricle, the most important points to define are the position of the apex and the diameter of the mitral annulus, since the left ventricular long axis and the mitral ring diameter become proxies for long- and short-axis function. The geometric contribution to LV volumes of the curvature of the septum and the free wall is small. The "AutoEF"-method (syngo^®^AutoEF, Siemens Medical Solutions) exploits the use of a large database describing the variability in the position of the contour of the left ventricle. Thus, in difficult-to-image patients, it fills in data where the endocardial border is not visualized. However, in a number of patients manual interaction of the operator is required. In the present software version 1.0, AutoEF analyses only Siemens Sequoia DICOM images. In version 2.0, now available, "AutoLeftHeart", DICOM images from different ultrasound vendors can be analysed.

Three-dimensional (3D) volumetric calculations have been hailed as the future gold standard of quantitation. 3D may have fewer geometric assumptions for calculating volumes, but is no less sensitive to poor acoustic windows than standard tomographic 2D views [16].

The aim of this study was to compare an automatic computerized algorithm using an adaptive appearance model of the left ventricle with conventional echocardiographic methods to estimate left ventricular volumes and ejection fraction in clinical practice.

## Methods

### Study population

Sixty patients (19 women and 41 men, age 61 ± 10, height 174 ± 11 cm, weight 84 ± 16 kg), with known or suspected coronary artery disease scheduled for MPI, were enrolled in the study. All patients were in sinus rhythm, which, however, did not constitute a criterion for inclusion. Twenty-four had a history of previous myocardial infarction and 28 had earlier been revascularized. Thirty-three were smokers or ex-smokers. The only exclusion criterion was unwillingness to participate in the study. One patient had to be excluded due to technical problems with the images. Pharmacologic treatment was held constant. For each patient two-dimensional echocardiography was performed within one hour of MPI at rest.

### Ethics and consent

The study complied with the Declaration of Helsinki and with agreements on Good Clinical Practice. Approval was obtained by the Regional Ethical Review Board in Linköping. All subjects gave written informed consent.

### Protocol

Ejection fraction and left ventricular volumes were determined with three echo-based methods and MPI. Five experienced readers (two certified by the accreditation procedure of the European Association of Echocardiography) and two novice readers (cardiology fellows early in their echo-training) were asked to quantify LVEF in each patient with three methods: (1) manual biplane Simpson (manual Simpson), (2) by applying the automatic software (AutoEF) in two apical orthogonal planes, with manual correction if needed (corrected AutoEF) and (3) visual assessment of LVEF(%) in four different categories (see below). In addition, one investigator analysed all studies without manually correcting the delineation by the AutoEF software (uncorrected AutoEF). Ten patients were randomly selected for assessment of intra- and interobserver variability. These patient studies were included twice, at random, in the studylist, to avoid bias. All images were anonymized. For measurements, anonymized DICOM-images were reloaded on the scanner, where manual Simpson and corrected AutoEF were performed. The image quality (sharpness of the endocardial border) as well as an estimate of ejection fraction was assessed visually. The time required for analysis of LVEF using the three methods was recorded. For both AutoEF analyses, the clock was started when the software was activated and stopped when the study report was opened and printed. For biplane Simpson, the clock was started when the study was opened and stopped when the print button was activated. Study anonymization was repeated between sessions that took place with a three week interval in order to minimize investigator bias. All investigators were blinded to the results of the isotope study.

### Echocardiography

Echocardiographic imaging was performed by four experienced operators (three technicians, one physician) with a Sequoia C512 (Siemens Acuson, Mountain View, California) using a broadband transducer (4V1c) operating in harmonic imaging mode. Clips of three consecutive beats in the apical 4-, 2- and 3-chamber views were stored digitally. The most representative beat in each view was selected for each patient. Image quality, defined as the extent of visualisation of the endocardium, was assessed by the readers in three groups: excellent (1), when all 12 segments of endocardium from the two views were seen, suboptimal (2) when 1–3 segments and poor (3) when 4 or more segments were insufficiently visualized.

#### Manual planimetry using biplane Simpson's rule (manual Simpson)

The endocardial border was manually traced in the apical 4- and 2-chamber views in end-diastole and end-systole. The ejection fraction was calculated by the computer software from volumes obtained by the summation of a stack of elliptical discs at end-diastole and end-systole, respectively.

#### AutoEF with and without manual correction

**Figure 1:**
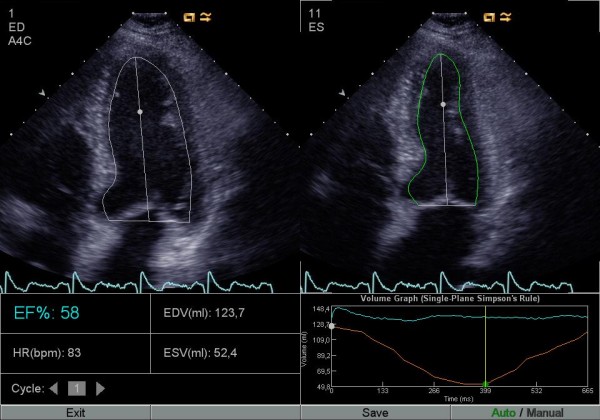
**AutoEF before and after manual correction**. The same images as Figure 1 after manual correction of the endocardial border by the operator. This panel of images shows an example of underestimation of the length of the longaxis of the left ventricle by the software.

**Figure 2:**
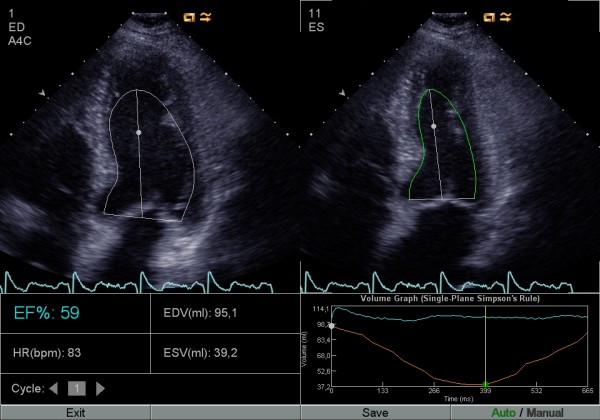
**AutoEF before and after manual correction**. Four-chamber view with automatic delineation of the endocardial border in diastole and in systole by the software.

**Figure 3:**
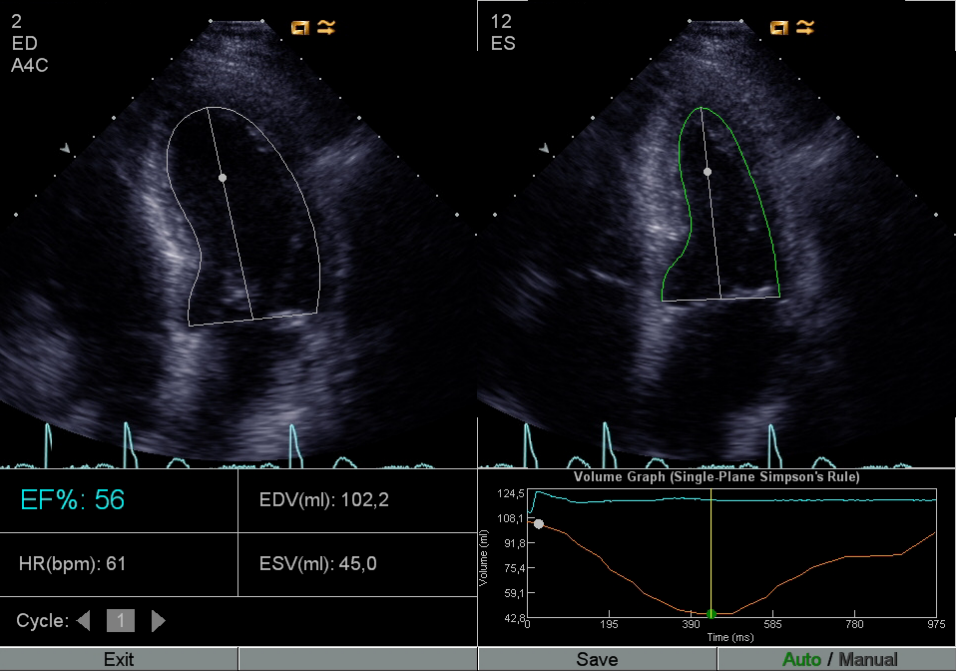
**AutoEF without need for manual correction**. Four-chamber view with automatic delineation of the endocardial border in diastole and in systole by the software. This is an example of a patient study that did not need manual correction.

Digitally stored apical 4- and 2-chamber views (identical with the ones used for manual Simpson) were analyzed with the automatic software (AutoEF) calculating LVEF, also using the biplane Simpson's formula. The investigators were allowed to manually correct the suggested delineation of the LV contour by click-and-drag (corrected AutoEF, Figures 1, 2 and 3), when needed. In addition, one investigator analysed all the studies with the automatic software without manually correcting the suggested delineation (uncorrected AutoEF).

#### Visual assessment of LVEF

Left ventricular function was visually assessed in four categories: normal (EF > 55%) (1), mildly impaired (EF 45–54%) (2), moderately impaired (30–44%) (3), and severely reduced (< 30%) (4) [5].

### Myocardial perfusion imaging (MPI)

Gated myocardial SPECT was performed using a two-day stress/rest protocol. Rest images were obtained using 8.6 MBq 99mTc-tetrofosmin/kg bodyweight. Supine gated SPECT images were acquired 45–60 minutes after the injection. The acquisitions were made on a dual-detector gamma camera (ECAM Siemens Medical Systems Inc) with a low energy high resolution collimator using 64 projections over 180° (right anterior oblique 45° to left posterior oblique 45°), 30 s per projection. A 19% window was asymmetrically placed (129–155 keV) on the 140 keV peak, asymmetry 2%. The gated and ungated data were separately reconstructed on a Hermes Medical Solutions (Stockholm, Sweden) workstation. Prefiltering with a Butterworth filter (cut off 0.8/cm, order 10) was applied followed by filtered back projection. No scatter- or attenuation correction was applied. The reconstructed transaxial images were manually realigned along the cardiac long axis. The short axis slices at rest were then processed with the automatic software package QGS (Cedars-Sinai Medical Center, Los Angeles, CA, USA) to calculate LV volumes and global LVEF.

### Statistical analysis

All statistical analyses were performed using SPSS 16.0 (SPSS Inc.). Paired and unpaired 2-tailed Students' t-tests were used along with ANOVA (followed by Duncans test in case of significance) and Pearson correlation coefficient as well as chi-square, when appropriate. Accuracy was evaluated with bias and limits of agreement (± 1.96 SD) determined from a Bland-Altman (B-A) analysis [17]. Intra-and interobserver variability of LVEF was expressed as standard error of a single determination (S_method_) using the formula, first proposed by Dahlberg [18]:

S_method _= √(∑d_i_^2^/(2n)),

where d_i _is the difference between the i:th paired measurement and n is the number of differences. S_method _was also expressed as % of over all means. Single measure intraclass correlation coefficient (ICC) was also used to express interobserver variability. ICC assesses rating reliability by comparing the variability of different ratings of the same subject with the total variation across all ratings and all subjects. Kappa measure of agreement was used to compare the estimated LVEF categories of visual assessment (EF ≥ 55%, 45–54%, 30–44%, and < 30%) and corresponding categorial values categorized from AutoEF, Manual Simpson and MPI.

## Results

### Left ventricular volumes and LVEF

#### Quality assessments and time required for the echocardiographic investigations

Echocardiographic imaging was possible in all patients. Image quality was rated excellent (< 2) in 26/59, suboptimal in 14/59 and poor (> 2.5) in 19/59 (average from 5 experienced readers). The time required for analysis was for uncorrected AutoEF 79 ± 5 s, for corrected AutoEF 159 ± 46 s, for manual Simpson 177 ± 66 s and for visual assessment 33 ± 14 s. The time differences between all the methods were significant (p from < 0.001 to 0.015) with visual assessment and uncorrected AutoEF being fastest.

#### Differences between the two AutoEF methods and manual Simpson

**Figure 4:**
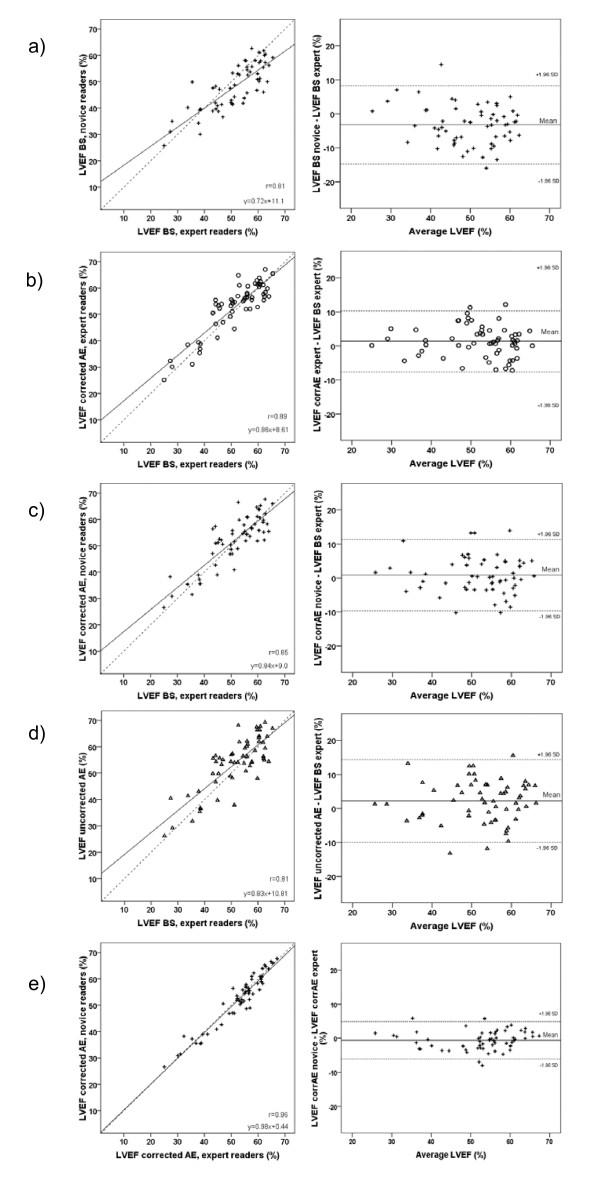
**Echocardiographic estimation of left ventricular ejection fraction**. Correlation analysis comparing the various echocardiographic estimations of LVEF with expert manual biplane Simpson (left panels) and corresponding Bland-Altman plots of the difference between them (right panels) a) the manual Simpson method by the novices, b) the corrected AutoEF method by the expert readers, c) the corrected AutoEF method by the novices and d) the uncorrected AutoEF method. Finally, e) displays the correlation between corrected AutoEF performed by either experts (horizontal line) or novices (vertical line). BS = manual Simpson, LVEF = left ventricular ejection fraction

Ejection fraction agreed well between the echocardiographic methods. It was for corrected AutoEF 53 ± 10% and for manual Simpson 51 ± 11%, Table 1. Manual Simpson was significantly lower than the other methods (p = 0.001–0.028; including myocardial scintigraphy; see below), but no differences were seen between the other methods. Pearson correlation coefficient between corrected AutoEF and manual Simpson was 0.89 and limits of agreement of 9.0% (Table 2 and Figure 4). For uncorrected AutoEF, ejection fraction was 54 ± 10% with a correlation coefficient of 0.81 compared with manual Simpson, limits of agreement 12.1%. Regardless of image quality, there were no differences between AutoEF (corrected and uncorrected) and manual Simpson. End-diastolic and end-systolic volumes were slightly lower for the two AutoEF methods compared with manual Simpson, with a bias between 2.6–5.8 mL/m^2 ^(Table 2).

**Table 1:** Group results for the transthoracic echocardiographic and MPI variables

	**Corrected AutoEF** **n = 59** (5 readers)	**Uncorrected AutoEF** **n = 59** (1 reader)	**Manual Simpson** **n = 59** (5 readers)	**MPI** **n = 59** (1 reader)
**LVEF (%)**	53 ± 10	54 ± 10	51 ± 11	52 ± 12
**EDV/BSA (mL/cm2)**	53 ± 18	51 ± 16	56 ± 20	67 ± 26
**ESV/BSA (mL/cm2)**	26 ± 14	24 ± 13	28 ± 15	35 ± 23
**Time for analysis (s)**	174 ± 43	79 ± 5	190 ± 64	
**Range (s)**	96–417	68–90	100–615	

Mean ± SD

BSA = body surface area, EDV = End-diastolic volume, ESV = End-systolic volume, LVEF = Left ventricular ejection fraction, MPI = myocardial perfusion imaging.

**Table 2:** Correlation analysis and limits of agreement (Bland-Altman analysis) between the echocardiographic methods based upon the experience of the readers.

	**Expert (n = 5)**		**Novice (n = 2)**		**Expert (n = 1)**	
	Limits of agreement	r	Limits of agreement	r	Limits of agreement	r
**LVEF (%):**						

**cAutoEF vs BS expert**	1.4 ± 9.0	0.89	0.8 ± 10.5	0.85		
**AutoEF vs BS expert**					2.2 ± 12.1	0.81
**BS novice vs BS expert**			-3.2 ± 11.4	0.81		
**cAutoEF novice vs expert**			-0.6 ± 5.5	0.96		

**EDV/BSA (mL/cm2):**						

**cAutoEF vs BS expert**	-3.3 ± 13.0	0.94	-2.3 ± 13.1	0.94		
**AutoEF vs BS expert**					-5.8 ± 14.4	0.86
**BS novice vs BS expert**			18.8 ± 22.1	0.87		
**cAutoEF novice vs expert**			1.0 ± 5.1	0.99		

**ESV/BSA (mL/cm2):**						

**cAutoEF vs BS expert**	-2.6 ± 7.8	0.97	-1.8 ± 8.0	0.97		
**AutoEF vs BS expert**					-4.3 ± 9.3	0.95
**BS novice vs BS expert**			12.1 ± 12.4	0.93		
**cAutoEF novice vs expert**			0.8 ± 3.5	0.99		

Limit of agreement is calculated as bias ± 1.96

SD BS = manual biplane Simpson, BSA = body surface area, cAutoEF = corrected AutoEF, EDV = End-diastolic volume, ESV = End-systolic volume, LVEF = Left ventricular ejection fraction

#### Differences between the echocardiographic methods and MPI

Ejection fraction for MPI was 52 ± 12%. Values > 65% were approximated to 65% (four patients) because of the partial volume effect [19]. Left ventricular volumes, normalized to body surface area, were largest for MPI, smallest for corrected and uncorrected AutoEF and intermediate for manual Simpson (Table 1).

**Figure 5:**
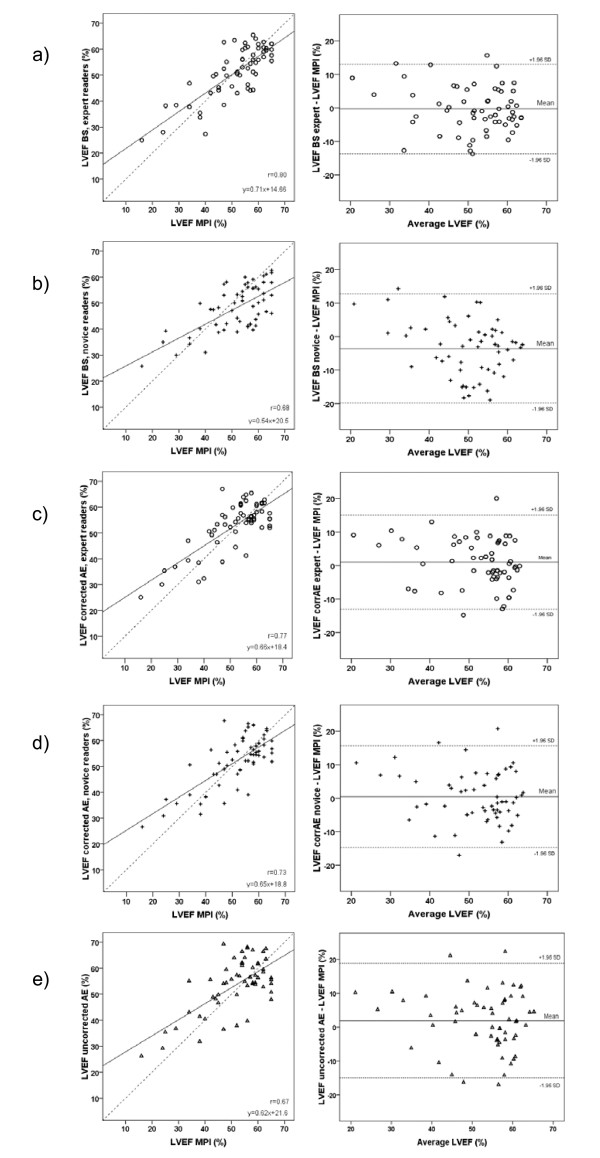
**Estimation of left ventricular ejection fraction – echo versus MPI**. Correlation analysis comparing the various echocardiographic estimations of LVEF with that of myocardial gated perfusion imaging (left panels) and corresponding Bland-Altman plots of the difference between them (right panels) a) the manual Simpson method by the expert readers, b) the manual Simpson method by the novices, c) the corrected AutoEF method by the expert readers, d) the corrected AutoEF method by the novices and e) the uncorrected AutoEF method. BS = manual Simpson, MPI = myocardial perfusion imaging, LVEF = left ventricular ejection fraction

Correlation analysis between corrected AutoEF, uncorrected AutoEF and manual Simpson (expert group) versus MPI showed coefficients of r = 0.77, 0.67 and 0.80, respectively, and limits of agreement 14.0%, 16.9% and 13.4% (LVEF units), respectively (Table 3 and Figure 5). The corresponding correlation coefficients were for the novices r = 0.73 and r = 0.68 for corrected AutoEF and manual Simpson, respectively. Compared with MPI, volumes from corrected AutoEF and manual Simpson were generally lower for experienced readers. On the contrary, the two novices had somewhat larger volumes using manual Simpson than the reference volumes from MPI (Table 3).

**Table 3:** Correlation analysis and limits of agreement (Bland-Altman analysis) between the echocardiographic methods and MPI based upon the experience of the readers.

	**Expert (n = 5)**		**Novice (n = 2)**		**Expert (n = 1)**	
	Limits of agreement	r	Limits of agreement	r	Limits of agreement	r
**LVEF (%):**						

**cAutoEF vs MPI**	1.0 ± 14.0	0.77	0.4 ± 15.2	0.73		
**BS vs MPI**	-0.3 ± 13.4	0.80	-3.6 ± 16.3	0.68		
**AutoEF vs MPI**					1.9 ± 16.9	0.67

**EDV/BSA (mL/m2):**						

**cAutoEF vs MPI**	-14.1 ± 25.2	0.92	-13.1 ± 27.1	0.89		
**BS vs MPI**	-10.8 ± 25.6	0.89	8.0 ± 30.9	0.80		
**AutoEF vs MPI**					-16.6 ± 28.5	0.87

**ESV/BSA (mL/m2):**						

**cAutoEF vs MPI**	-8.7 ± 22.8	0.95	-7.9 ± 24.1	0.92		
**BS vs MPI**	-6.2 ± 21.6	0.93	6.0 ± 23.4	0.87		
**AutoEF vs MPI**					-10.4 ± 25.8	0.90

Limit of agreement is calculated as bias ± 1.96

SD BS = manual biplane Simpson, BSA = body surface area, cAutoEF = corrected AutoEF, EDV = End-diastolic volume, ESV = End-systolic volume, LVEF = Left ventricular ejection fraction MPI = myocardial perfusion imaging

#### Visual assessment of ejection fraction

The estimated categories of LVEF(%) by visual assessment (EF ≥ 55%, 45–54%, 30–44%, and < 30%) compared to corresponding categorical values calculated for AutoEF, manual Simpson, and MPI (Table 4) showed kappa measures of agreement of 0.47, 0.44, and 0.52, respectively for expert readers. For comparison, corresponding value for AutoEF and Manual Simpson was 0.53.

**Table 4:** Comparison of visual assessment, AutoEF, Manual Simpson and myocardial perfusion imaging (MPI), expert readers.

		**Visual assessment of LVEF(%)**				
		
	**LVEF%**	**≥ 55**	**45–54**	**30–44**	**< 30**	**Total**
**AutoEF**						
	**≥ 55**	90	42	10	1	143
	**45–54**	31	42	25	1	99
	**30–44**	1	9	22	11	43
	**< 30**	0	0	4	6	10

**Manual Simpson**						
	**≥ 55**	81	41	7	1	130
	**45–54**	31	37	18	2	88
	**30–44**	10	15	30	9	64
	**< 30**	0	0	6	7	13

**MPI**						
	**≥ 55**	99	45	6	0	150
	**45–54**	21	38	24	2	85
	**30–44**	2	10	19	9	40
	**< 30**	0	0	12	8	20

**Row**	**Total**	122	93	61	19	295

Note that there are five readings per patient for each method. The numbers of the single reader in MPI has been multiplied by 5.

### Intra- and interobserver variability

For uncorrected AutoEF, the reproducibility was 100% and not analysed further. Intraobserver variability (S_method_) for corrected AutoEF was for the expert readers 2.2 LVEF percentage points (4.7%) and for the novices 2.9 (6.4%). Corresponding values for manual Simpson was for experienced readers, 3.5 LVEF percentage points (7.7%) and for novices 6.2 (14.6%). The difference in intraobserver variability between corrected AutoEF and manual Simpson was significant for both the experts (p = 0.004) and the novices (p = 0.008). As expected, experienced readers had significantly lower variability (p < 0.001).

The interobserver variability for the echocardiographic methods was analysed both with the S_method _by Dahlberg and the intraclass correlation coefficient (ICC). Highest ICC was found for corrected AutoEF (0.88 for experienced readers and 0.81 for novices) with corresponding values of S_method _of 3.5 (6.7%) and 4.4 (8.5%), respectively. ICC was lower for manual Simpson, especially novices (corresponding values 0.74 and 0.21) with values of S_method _of 6.0 (11.6%) and 12.4 (25.7%), respectively, which demonstrates the low interrater agreement for the novices. Interobserver variability for MPI was calculated with the Dahlberg formula as 1.0 LVEF percentage points (only 3 of the 59 patients differed between the two expert readers evaluating MPI). Corresponding value for visual assessment was 0.48 categorized steps for the five experts.

## Discussion

### Main findings of the study

**Figure 6:**
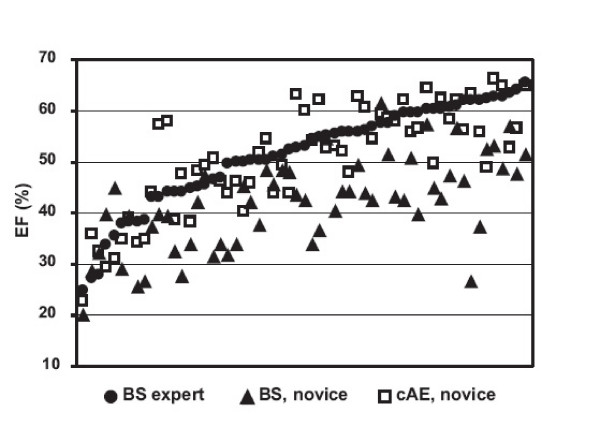
**Effect of computer-assisted measurements on novice performance**. Plot of left ventricular ejection fraction (LVEF%), ranked after increasing values of the expert manual Simpson (filled circles). Values in line above and below BS expert belongs to the same patient and show corresponding values for novice manual Simpson (filled triangle) and novice corrected AutoEF (empty square). The smallest deviation from expert manual Simpson is seen for novice corrected AutoEF. BS = manual Simpson, MPI = myocardial perfusion imaging, LVEF = left ventricular ejection fraction

In this study, we show for the first time a reduced variability in the measurement of ejection fraction when experienced and novice readers use biplane AutoEF. Due to AutoEF, intra- and interobserver variability was reduced compared with manual biplane planimetry, especially for novice readers (Figure 6). The addition of manual corrections to AutoEF produced somewhat better estimates of volumes but not of LVEF. Without manual correction, the application of AutoEF on the scanner took on average 79s. It could be envisioned that this time might be further reduced with faster computer processing.

### Left ventricular ejection fraction from AutoEF compared with LVEF using manual biplane Simpson's rule

Manual delineation of the left ventricular contour using manual biplane Simpson's rule, by many considered to be the reference method of choice, displayed a larger variation in measured values than corrected AutoEF for the novices, but not for the experts (Table 2). To reduce this variation, the use of especially trained technicians in core labs has been suggested which, however, is unrealistic in clinical work [20]. Another avenue for improvement could be the use of computer based methods using learned pattern recognition and artificial intelligence such as AutoEF. Previous authors [21] found encouraging results using single plane AutoEF. Objections were voiced by Rahmouni et al, who, however, did not perform manual corrections of obviously erroneous delineations of the left ventricle [22]. They reported a low correlation between AutoEF and manual planimetry as well as between AutoEF and MRI, but did not show the correlation between planimetry and their gold standard MRI. The suboptimal performance of single plane AutoEF seems rather obvious for left ventricles with regional wall motion abnormalities. Results should improve with a biplane approach such as in our study. In contrast to other studies, we did not exclude patients on the basis of image quality. Those with poor image quality showed a similar agreement compared to MPI as those with good image quality. In our hands, this method seems to be able to reduce variation in the assessment of LVEF in clinical patients. Furthermore, in studies of this kind, it is necessary to correctly blind the image readers, to avoid bias, and to use the reference method in all patients. Both conditions were successfully applied which strengthens the results obtained.

### Which reference? Biplane Simpson or myocardial perfusion imaging?

In a comparison between methods, precision ("accuracy") is as important as a low random variation in measurements. Accuracy is dependent on the reference method used. A better agreement is expected if the reference method uses identical images, as in this study where AutoEF was compared with manual planimetry on echocardiographic images. Both echo-based methods use two heart beats for the calculation of LVEF, while SPECT uses information selected during an acquisition that takes on average 20 minutes. During this time period, the patient's heart rate may vary. The recorded values for heart rate were, however, very similar for echo and MPI. We selected gated SPECT as the reference because of availability, personal experience, ease of use and solid scientific documentation [23,24], even though magnetic resonance imaging by many is considered to be an absolute gold standard [25]. In line with previous publications [26], we found an underestimation of volumes determined with echocardiography but a good agreement for ejection fraction, compared with MPI.

### Can visual assessment classify the level of left ventricular dysfunction?

Is visual assessment a viable alternative to AutoEF for the determination of LVEF? Some studies [6] have reported enthusiastic positive experience of using eye-balling, even at a level of determining single percentage points of LVEF. McGowan, however, in a recent meta-analysis expressed a more guarded attitude [8]. Our study only assessed visual classification in four broad categories. Although visual assessment used in this manner performed in line with the other methods, our belief is that decisions regarding advanced cardiac treatments should preferably be based on quantification and not on qualitative visual assessment. In that respect, methods like AutoEF are of great value, especially for the novices.

### Training in echocardiography and the need for computer-aided support software

The two novices in this study had less than two months of formal echo training and had never before performed manual planimetry for ejection fraction. The large variation in their results for manual planimetry can be seen in Figure 4. However, the novices performed almost as well in their use of corrected AutoEF as the experienced group (p = n.s.). Manual Simpson showed a larger variation when used by novices compared to expert readers. We suggest that readers at an early stage of their training benefit the most from using computer-supported methods for the determination of LVEF.

### Limitations

The majority of the patients in this study had normal or slightly reduced LVEF. Only 15 of the 59 patients had LVEF < 45%. However, it is important to determine small changes in LVEF also close to the normal range, considering the use of echo for the monitoring of potentially cardiotoxic drugs. Myocardial gated SPECT (MPI), has some limitations mainly in the low frame rate used and the long acquisition time that produces a mean value for ejection fraction over 20 minutes. ECG-gated SPECT using 8 time frames has been shown to underestimate LVEF because of low frame rate as well as a tendency to overestimate high-normal LVEF values due to partial volume effects in small hearts. In this study we only corrected the supernormal LVEFs [19,27]. MPI is used as reference to show that our volumes and calculated ejection fraction results are plausible and was chosen because the patients in our study were referred for MPI which, as a by-product, gives us the ejection fraction measurement. MRI, being one gold standard for volume determinations, was not possble to perform in the present setting in these patients. At the time of the study we did not have access to 3D echo. Even if many echo labs nowadays buy 3D capability, 2D-based methods will prevail in a medium term perspective due to the large installed base of contemporary ultrasound equipment. A larger cohort of novice readers was difficult to achieve with the manpower available at the participating echo labs. Finally, AutoEF in our hands required manual corrections in most patients. A fully automated and faster method still awaits invention.

## Conclusion

A computer software using learned pattern recognition and artificial intelligence (AutoEF) applied on biplane apical echocardiographic views reduces the variation in measurements compared with manual planimetry, especially for less experienced readers, without increasing the time required. This should be valuable particularly in the follow-up of patients receiving potentially cardiotoxic treatment where small variations in measured LVEF may trigger changes in therapy.

## List of abbreviations

ANOVA: Analysis of Variance; B-A: Bland and Altman; BS: Biplane Simpson; DICOM: Digital Imaging and Communications in Medicine; ECG: ElektroKardioGram; ICC: Intraclass Correlation Coefficient; LV: Left Ventricle; LVEF: Left Ventricular Ejection Fraction; MAM: Mitral Annular Motion; MPI: Myocardial Perfusion Imaging; MRI: Magnetic Resonance Imaging; SD: Standard Deviation; SPECT: Single Photon Emission Computed Tomography; SPSS: Statistical Package for the Social Sciences.

## Competing interests

The authors declare that they have no competing interests.

## Authors' contributions

EM planned the study, investigated all patients, performed all measurements as well as all analyses and the main part of writing the manuscript. LB participated in the statistical analysis of the results and in writing of the manuscript. EN performed some measurements and participated in the writing of the manuscript. LL performed some measurements and participated in the writing of the manuscript. JO planned the study together with EM, was responsible for the evaluation of the scintigraphic part, took part in statistical analyses and in writing of the manuscript. JE participated in the planning of the study, performed all measurements and took a major part in the writing of the manuscript. All authors have read and approved the final manuscript.
